# Molecular modelling and docking analysis of pleurocidin (an antimicrobial peptide) like peptides with enterotoxin H from Klebsilla pneumonia

**DOI:** 10.6026/97320630015838

**Published:** 2019-12-17

**Authors:** Giridharan Bupesh, Manickam Sivaraman Nandini, Sakthivel Vasanth, Tharumasivam Siva Vijayakumar, Chinnaiah Amutha, Kaliyaperumal Prabhu, Vellingiri Balachnadar

**Affiliations:** 1 Research and Development Wing, Sree Balaji Medical College and Hospital, BIHER, Chrompet, Chennai 600044, India; 2 Department of Animal Science, School of Life Sciences, Bharathidasan University, Tiruchirappalli, India; 3 Department of Biotechnology, Srimad Andavan College, Tiruchirappalli, India; 4 Department of Animal Behavior and Physiology, Madurai Kamaraj University, Palkalaiperur, Madurai, India; 5 Department of Anatomy, Sree Balaji Medical College and Hospital, BIHER, Chrompet, Chennai-600044, India; 6 Department of Microbiology, Sree Balaji Medical College and Hospital, BIHER, Chrompet, Chennai600044, India; 7 Department of Human genetics and molecular biology, Bharathiyar University, Coimbatore, Tamilnadu, India

**Keywords:** Enterotoxin H protein, Klebsilla pneumonia, pleurocidin, anti microbial peptide, modeling, docking

## Abstract

Enterotoxin H is a key molecular target for replication and establishment of Klebsilla pneumonia in the host. Therefore, it is of interest to study the interaction of enterotoxin H with pleurocidin like peptides using molecular modelling (template PDB ID: 1YCE), Lig-Plot (ligand construction) and docking tools for therapeutic consideration. The hydrophobic pocket and the active site residues (Val 13, Met 16, Gly 25, Ala 25, and Ile 28) were identified using Cast P, Molegro and Sitehound tools. Docking results show that the pleurocidin like peptides interacts with the active sites of enterotoxin H with 300.96 docking score with optimal binding features.

## Background

Enterotoxin H is a key molecular target for replication and establishment of Klebsilla pneumonia in the host [1-3]. They are associated with endophthalmitis and urinary tract infection (UTI) [4]. A detailed understanding of the molecular structure and function of Enterotoxin H is highly relevant [5-8]. Therefore, it is of interest to study the interaction of enterotoxin H with pleurocidin like peptides using molecular modelling (template PDB ID: 1YCE), Lig-Plot (ligand construction) and docking tools for therapeutic consideration. The use of molecular docking tools such as DOCK [9-11], FlexX [12], GOLD [13], and ICM [14] in drug discovery has become routine in recent years. The search methods and score functions of various docking tools are known [15]. We describe the optimal features that support pleurocidin like peptide interaction with Enterotoxin H from Klebsilla pneumonia.

## Methodology

### Target peptide sequence:

**Figure 1:**
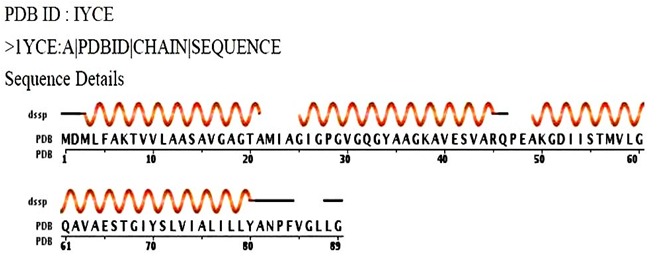
MALDI TOF peptide sequence from Clarias batrachus

The pleurocidin like peptide MALDI TOF sequence from Clarias batrachus is given below is shown in Figure 1.

### Protein template:

**Figure 2:**
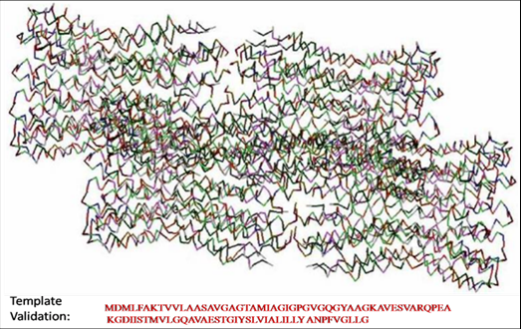
The 3D sstructure of template membrane protein (PDB ID: 1YCE) generated using the modeller software.

The 3D structure of the template membrane protein (Research Collaboration for Structural Biology (RCSB) Protein Data Bank (PDB) ID: 1YCE) is shown in Figure 2.

### Ligand data:

The ligand sequence data given below for enterotoxin H from K. pneumonia is downloaded from NCBI.

### Enterotoxin type H [Klebsiella pneumonia subsp. rhinoscleromatis ATCC 13884]:

>gi|262043668|ref|ZP_06016777.1| enterotoxin type H [Klebsiella pneumonia subsp. rhinoscleromatis ATCC 13884] MSGLTRKKIAVLELIRTCSEGVTSAEVMYSLGMSRSTVFFILDSLLKDNLIFRAHNETGRNSRRIYFPTAELAEKFSGKKIPMSKRESFFDSCRRHSKNYMITLLLRSAR QPPKEENQ 

### MODELLER software:

The MODELLER software package is used for homology or comparative modelling of protein 3D structures using default parameters [8,9].

### Ligplot:

The LIGPLOT program was used for showing the 2-D representation of protein-ligand interactions in standard PDB data format.

### GOLD - protein-ligand docking:

The GOLD protein ligand docking package was used for molecular docking analysis.

### SITEHOUND:

This tool identifies ligand binding sites by computing interactions between a chemical probe and a protein structure using PDB input data.

### Model validation:

Model validation was completed using the Ram Page server and CE.

## Results and Discussion:

**Figure 3:**
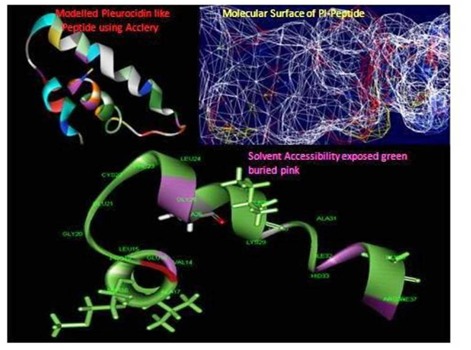
Solvent accessibility and surface features of the peptide.

The crystal structure of the membrane protein ATP synthase (PDB ID: 1YCE) is used as the template structure (Figure 1). The identity of the target and the template were screened to construct the model for the target pleurocidin like peptide using the protein modelling package modeller. A 40% sequence (40 %) similarity was found between target and the template. The red coloured alphabets in the alignment showed the similarity between the template and target where the conserved motif was identified (Figure 2). The solvent accessibility is one of the key factors that determines the ligand interaction and binding of the receptor – ligand complex (Figure 3). Red coloured side chains in the Figure 3 show the active solvent accessible layer.

**Figure 4:**
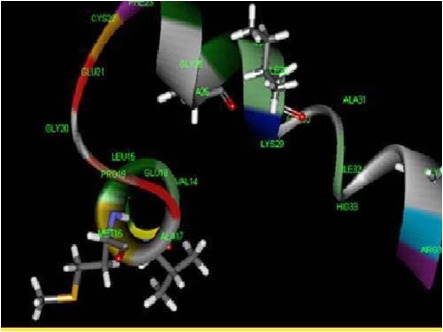
Visualization of predicted active site in enterotoxin H using Discovery Studio.

The modelled protein PL peptide was displayed in the Figure 3 which shows the superimposed secondary structure the α-helical patterns with extended sheet in the modelled structure. The exposed layer pink colour buried in the peptide chain shows the motif availed to access ligand structure (Figure 3). The catalytic active site in the ligand PLP binds the enterotoxin with the residues in the positions of Valine - 13, Methionine - 16, Glycine - 25, Alanine - 26 and Isoleucine - 28 predicted as the active residues as shown by Accelerys Discovery StudioTM (Figure 4).

**Figure 5:**
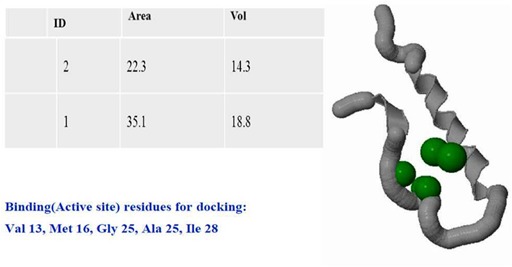
Binding site prediction of the peptide using CastP tool.

**Figure 6:**
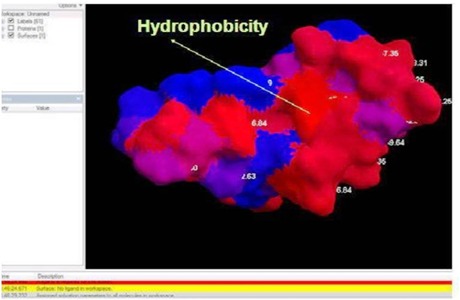
Prediction of hydrophobicity sites in the peptide using Molegro.

**Figure 7:**
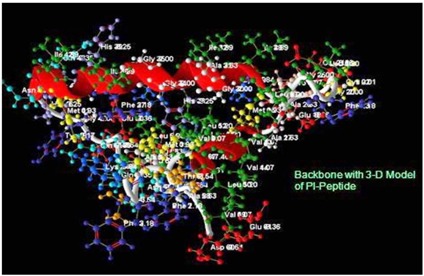
Backbone structure of the peptide using the Accelery's Protein Viewer.

This was further analyzed for the ion containing amino acid residues in the binding pocket using the tool cast-P one of online tool (Figure 5). Figure 5 indicate the aminoacid residue (binding Active site residues) for docking were four residues like Val 13, Met 16, Gly 25, Ala 25 and Ile 28. Hydrophobicity was a vital factor for structure activity relationship in binding. The Red colour buried layer in Figure 6 shows 50% hydrophobicity in the PL-peptide structure. The solid 3D-entitiy showed the highest hydrophobic interaction present in the structure, which facilitates the receipting activity on receptor-ligand complex of the PL-peptide and enterotoxin H complex. The RMSD (Root Mean Square Deviation) of the modelled structure is within acceptable limit.The modelled proteins were evaluated using threading to validate the constructed model PL-peptide. So the constructed structure was analyzed by the Ram page server and Swiss pdb viewer [16-18]. The Ram page server validated the structure with allowed number of aminoacid in the favoured region (above 94%). The PL peptide in Figure 7 shows 96% allowed region in the model. This indicates that the constructed PL-peptide model was well constructed and perfectly assigned in the structural and geometric entity. 

**Figure 8:**
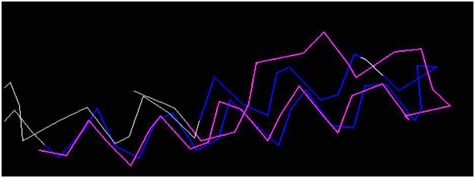
Combinatorial Extension and alignment of target peptide and template (PDB ID: 1YCE) with the Root mean square deviation (RMSD) of 2.6 Å.

**Figure 9:**
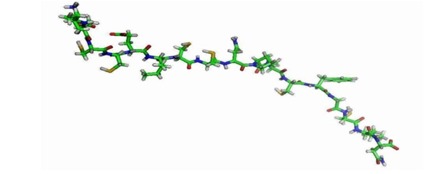
Three-dimensional structure of enterotoxin H ligand from K. pneumoniae using Ligplot.

The RMSD between template and target is 2.6 Å which was below rule 5 within accepted cut-off (Figure 8). Further, the alignment of the target and template identity to validate the structure was shown in Figure 7. The chain in the template and target showed the identical motif with a perfect model. The ligplot tool was used to show the enterotoxin H structure for the optimal preparation of the ligand enterotoxin-H. Thus, we used ligplot to show the enterotoxin H structure (Figure 9).

**Figure 10:**
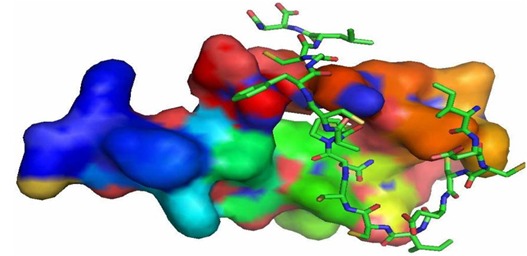
Docking of peptide with enterotoxin H using the Gold software.

The structure of target was developed using the template and docked using the gold docking software. The docking of the PL-peptide and enterotoxin H was well docked (Figure 10). The ligand enterotoxin H was bonded in the active site. The amino acid residues involved in the docking of the ligand enterotoxin H was analyzed using the cluster of site Hound web server were (VAL9, LEU10, VAL13, VAL14, ALA17, ALA26, ILE28, LYS29 and LEU 27) as shown in Table 1. The cluster coordinates and the total energy for docking in coordinates were obtained (-500) which shows good receipting energy levels - 318.728, -318.330, -254.794, -155.508, 155.238, -82.492, -66.544, -59.139, -20.309 (Table 2). After the docking the best ligand transformation energy was found as -7.50834 65.33267 20.29962, and the docking score was noted as -300.96 (Table 3). It is known that values below 1 are considered as good docking score in the Gaussian docking rules as shown in Table 4.

The antimicrobial peptide was targeted to produce a peptide therapeutic. Therefore, it is of interest to understand the PL-peptide structure and the receipting activity with the pathogenic toxin from Klebsiella pneumoniae. The PL-peptide was modelled and it was optimized for the docking process followed by virtual screening as described elsewhere [19,20]. It was found that glycosyl amines are suitable drugs to halt the growth of M. tuberculosis [21]. The modelling of PL-peptide by the modeller packages shows the three possible entities with values 157.9, 121.8 and 138.3. Data with the lowest value of 121.8 for conformation is used further as described by Kuntz et al. [9]. Alignment was performed for conserved motif to assess similarity between the target and the template. A 40% identity was found between template and target. Thus, the model was constructed using the template membrane protein with PDB ID: 1YCE.

The constructed model was evaluated by the ram page server and the combinatorial extension. The Ram page server validated the structure and reports the number of aminoacid in the favoured region (above 94%). Similarly, the number of allowed regions and the outlier region was expected as less than 3% and more than 1% the PL-peptide showed with an allowed region (2%) and outlier region (3.1%). This indicated that the constructed PL-peptide model was well modelled and perfectly assigned for structural and geometric analysis. The RMSD between template and target is 2.6 Å which was below allowed cut off limit. Then the modelled PL-peptide was docked with the ligand enterotoxin H receptor. The enterotoxin H receptor was protein toxin secreted by the Klebsiella pneumoniae. Hence, it is of interest to study the interaction between a peptide and the enterotoxin-H using docking tools. The PL-peptide is a peptide antibiotic against Klebsiella pneumoniae known by in vitro and in vivo studies in the mice. There is a strong evidence for receipting activity of PL-peptide with enterotoxin H. 

The important docking parameters such as solvent accessibility, binding site prediction, hydrophobicity were analyzed in the receptor, the ligand and optimized for the docking as described elsewhere [22-28]. Active binding sites were predicted using the Cast P tool for studying the receptor protein ligand interactions [29-31]. Similarly, the active site binding sites of PL-peptide were predicted using the AcceleryTM and Cast P web tools. The amino acid residue such as Val 13, Met 16, Gly 25, Ala 25 and Ile 28 were predicted in the pocket of binding site. In the PL-peptide the Ligand enterotoxin H were optimized to find the active site of ligand enterotoxin- H. The site hound web tool was used for predicting active sites in the ligand (VAL9, LEU10, VAL13, VAL14, ALA17, ALA26, ILE28, LYS 29 and LEU 27). The cluster coordinates and the total energy for docking were calculated using the gold package as -500 which shows good receipting energy level such as 318.728,-318.330,-254.794,-155.508,-155.238,-82.492,-66.544,-59.139, 20.309. The best ligand transformation energy was noted as - 7.50834 65.33267 20.29962, and the docking score was -300.96. Thus, we report data to support the optimal binding of PL-peptide with the enterotoxin H from K. pneumonia for further consideration.

## Conclusion

We report the molecular modelling and docking analysis data (300.96 docking score and - 7.50834 ligand transformation energy) of pleurocidin like peptide (an antimicrobial peptide) with enterotoxin H from Klebsilla pneumonia for further consideration as a therapeutic agent.

## Figures and Tables

**Table 1:** Enterotoxin H active site amino acids with cluster number used in molecular docking

Cluster Number	Amino acid Residues	
1	VAL 9	LEU 10
1	VAL 13	VAL 14
1	ALA 17	ALA 26
1	ILE 28	LYS 29
1	LEU 27	

**Table 2:** Ligand binding site data of pleurocidin like peptide

Cluster	Total Energy	Cluster Center Coordinates (x, y, z)			Cluster Volume
1	-318.728	-6.121	8.713	32.918	29
2	-318.33	-3.317	9.975	40.22	28
3	-254.794	3.397	16.216	35.589	23
4	-155.508	-17.28	5.844	37.409	14
5	-155.238	-10.385	0.648	37.053	15
6	-82.492	-10.945	17.735	32.253	8
7	-66.544	-21.281	14.542	33.702	7
8	-59.139	-4.56	18.053	41.417	6
9	-20.309	1.37	4.04	34.299	2

**Table 3:** Ligand transformation energy for docking of pleurocidin like peptide with enterotoxin H

S. No	Score	Pen	Area	ACE	Hydrophobicity	Ligand transformation
1	10070	-2.98	1253.4	-300.96	884.39	0.45739 -032866 -2.12321 -7.50834 65033267 20.29962
2	10046	-3.19	1643.8	-448.13	1061.02	1.30368 0.25697 -2.21050 -0.91653 28055330 -12028167
3	10022	-3.05	1775.8	-534.82	1183.72	1.02629 -0.95650 -1019755 -38.62009 58.06316 42050753
4	9880	-3.2	1457.1	-546.37	1042.23	3.10029 -1.21276 -1.23431 24.65437 19.61736 81.45148
5	9752	-2.82	1355.3	-409.39	955.27	-2.96609 -0.42663 -3.06663 21.44037 -13.55722 72.42266
6	9600	-3.4	1534.2	-306.61	992.78	1.67358 0.18419 -2032517 11.27280 19.85314 -1109149
7	9572	-3.73	1576.5	-579.83	1189.57	-2.91016 -0.30529 -1.06260 -3024514 56081124 71.27678
8	9410	-2.6	1176.3	-190.64	898.08	1.50635 0.43557 -1.93094 -4.29929 17.79129 -15.74896
9	9342	-2.92	1293.9	-646.41	897.5	-1.87840 0.35531 -0.68622 -11.16159 25.16533 86.13409
10	3914	-3.51	1536.5	-366.04	1123.13	1.26512 -0.78200 -1.21678 -34.57376 61.45337 29.62496
11	9254	-3.31	1474.1	-369.93	1149.21	-1.32643 -0.16566 2.07876 12.04785 -4.20350 82.76295

**Table 4:** Optimized parameters for docking of pleurocidin like peptide with enterotoxin H

Program	Parameters
ACE Energy Term Weight (Str)	1
COM distance Term Weight (Str)	1.07
HBEnergy Term Weight (Str)	1
Attr VdWEnergy Term Weight (Str)	1.01
Baseparams(Str)	4.013.02
Clusterparams(Str)	0.142.04.0
Confprob Energy Term Weight(Str)	0.1
Desolvationparams(Str)	500.01.0
elecEnergy Term Weight (Str)	0.1
EnergyDistCutoff (Str)	6
LigandGrid (Str)	0.5 6.0 6.0
LigandMs (Str)	Enterotoxin.pdb.ms
LigandPdb (Str)	Enterptoxin.Pdb
LigandSeg (Str)	10.0 20.0 1.5 10 10
Log-file (Str)	Patch dock.log
Log-level (Str)	2
MatchAlgorithm (Str)	1
matchingParams (Str)	1.5 1.5 0.4 0.5 0.9
piStackEnergyTermWeight (Str)	0
proLib (Str)	/specific/a/home/cc/cs/ppdock/webserver/patchDock/bin/chem.lib
radiiScaling (Str)	0.8
ReceptorGrid (Str)	0.5 6.0 6.0
receptorMS (Str)	Defense.pdb.ms
Receptorpdb (Str)	Defence.pdb
receptorSeg (Str)	10.0 20.0 1.5 10 10
repVdWEnergyTermWeight (Str)	0.5
ScoreParams (Str)	0.3 -5.0 0.5 0.00.01500 -8-4 01 0
ScoreParams (Str)	0.3 -5.0 0.5 0.00.0 1500-8-4010
vdWTermType (Str)	1+

## References

[1] Rudenko NV (2018). J Food Drug Anal.

[2] Sato'o Y (2015). Appl Environ Microbiol..

[3] Liu Y (2014). Toxins (Basel)..

[4] Lis E (2012). J Food Prot..

[5] Saline M (2010). Biomol NMR Assign..

[6] Sakai F (2008). J Food Prot..

[7] Ruzickova V (2008). Int J Food Microbiol..

[8] Jorgensen HJ (2005). FEMS Microbiol Lett.

[9] Kuntz ID (1982). Journal of Molecular Biology.

[10] DesJarlais RL (1988). Journal of Medicinal Chemistry.

[11] Rarey M (1996). Journal of Molecular Biology.

[12] Jones G (1997). Journal of Molecular Biology.

[13] Abagyan R (1994). Journal of Computational Chemistry.

[14] Taylor RD (2002). Journal of Computer-Aided Molecular Design.

[15] Boeckmann B (2003). Nucleic Acids Research.

[16] Berman HM (2000). Nucleic Acids Research.

[17] Arnold K (2006). Bioinformatics.

[18] Fields S, Song O (1989). Nature.

[19] Halperin I (2002). Proteins.

[20] Srivastava SK (2005). Nucleic Acids Research.

[21] Cheng T (2012). The AAPS Journal.

[22] Cavasotto CN, Orry AJ (2007). Current Topics in Medicinal Chemistry.

[23] Carlsson J (2008). Journal of Medicinal Chemistry.

[24] Barrett T (2013). Nucleic Acids Research.

[25] De Angelis M, Gobbetti M (2004). Proteomics.

[26] Nicola G (2008). Journal of Computational Biology.

[27] Stehn JR (2013). Cancer Research.

[28] Bupesh G (2014). International Journal of Drug Development and Research.

[29] Santhi MP (2016). Indian Journal of Medical Research and Pharmaceutical Sciences.

[30] Jai Prabhu (2016). International Journal of Pharmaceutical Sciences and Research.

[31] Vennila S (2014). Bioinformation.

